# Nationwide insecticide resistance status and biting behaviour of malaria vector species in the Democratic Republic of Congo

**DOI:** 10.1186/s12936-018-2285-6

**Published:** 2018-03-27

**Authors:** Francis Wat’senga, Emile Zola Manzambi, Andre Lunkula, Roger Mulumbu, Tania Mampangulu, Neil Lobo, Allison Hendershot, Christen Fornadel, Djenam Jacob, Mame Niang, Ferdinand Ntoya, Tamfum Muyembe, Joris Likwela, Seth R. Irish, Richard M. Oxborough

**Affiliations:** 1 National Institute of Biomedical Research, PO Box 1192, Kinshasa, Democratic Republic of the Congo; 2 National Malaria Control Programme, Kinshasa, Democratic Republic of the Congo; 3 321 Galvin Life Science Center, University of Notre Dame, Notre Dame, IN 46556 USA; 4 US President’s Malaria Initiative, US Agency for International Development, Washington, DC, USA; 5 PMI Africa Indoor Residual Spraying Project, Abt Associates 4550 Montgomery Ave, Suite 800 North, Bethesda, MD 20814 USA; 6 US President’s Malaria Initiative, Centers for Disease Control and Prevention, Kinshasa, Democratic Republic of the Congo; 7 US President’s Malaria Initiative, US Agency for International Development, Kinshasa, Democratic Republic of the Congo; 8 US President’s Malaria Initiative and Entomology Branch, Centers for Disease Control and Prevention, 1600 Clifton Road NE, Atlanta, GA 30329 USA

## Abstract

**Background:**

Globally, the Democratic Republic of Congo (DRC) accounted for 9% of malaria cases and 10% of malaria deaths in 2015. As part of control efforts, more than 40 million long-lasting insecticidal nets (LLINs) were distributed between 2008 and 2013, resulting in 70% of households owning one or more LLINs in 2014. To optimize vector control efforts, it is critical to monitor vector behaviour and insecticide resistance trends. Entomological data was collected from eight sentinel sites throughout DRC between 2013 and 2016 in Kingasani, Mikalayi, Lodja, Kabondo, Katana, Kapolowe, Tshikaji and Kalemie. Mosquito species present, relative densities and biting times were monitored using human landing catches (HLC) conducted in eight houses, three times per year. HLC was conducted monthly in Lodja and Kapolowe during 2016 to assess seasonal dynamics. Laboratory data included resistance mechanism frequency and sporozoite rates. Insecticide susceptibility testing was conducted with commonly used insecticides including deltamethrin and permethrin. Synergist bioassays were conducted with PBO to determine the role of oxidases in permethrin resistance.

**Results:**

In Lodja, monthly *Anopheles gambiae* s.l. biting rates were consistently high at > 10 bites/person/night indoors and outdoors. In Kapolowe, *An. gambiae* s.l. dominated during the rainy season, and *Anopheles funestus* s.l. during the dry season. In all sites, *An. gambiae* and *An. funestus* biting occurred mostly late at night. In Kapolowe, significant biting of both species started around 19:00, typically before householders use nets. Sporozoite rates were high, with a mean of 4.3% (95% CI 3.4–5.2) for *An. gambiae* and 3.3% (95% CI 1.3–5.3) for *An. funestus. Anopheles gambiae* were resistant to permethrin in six out of seven sites in 2016. In three sites, susceptibility to deltamethrin was observed despite high frequency permethrin resistance, indicating the presence of pyrethroid-specific resistance mechanisms. Pre-exposure to PBO increased absolute permethrin-associated mortality by 24%, indicating that resistance was partly due to metabolic mechanisms. The *kdr*-1014F mutation in *An. gambiae* was present at high frequency (> 70%) in three sites (Kabondo, Kingasani and Tshikaji), and lower frequency (< 20%) in two sites (Lodja and Kapolowe).

**Conclusion:**

The finding of widespread resistance to permethrin in DRC is concerning and alternative insecticides should be evaluated.

**Electronic supplementary material:**

The online version of this article (10.1186/s12936-018-2285-6) contains supplementary material, which is available to authorized users.

## Background

The Democratic Republic of Congo (DRC) is the second largest country in Africa and consists of several ecological zones, including large areas of tropical savannah and equatorial climate where significant rainfall occurs for more than 9 months a year and temperatures remain high. DRC has suffered through underdevelopment and conflict which has contributed to a UN human development index ranking of 176 out of 188 nations in 2015 [1]. The combination of ideal climate for *Anopheles gambiae* malaria vectors and underdevelopment have led to a particularly high malaria burden, with DRC accounting for 9% of global malaria cases and 10% of deaths [2]. According to the 2007 Demographic and Health Indicator Survey (DHIS), only 9% of households had one or more insecticide-treated nets (ITNs) [3]. Since then, significant progress has been made, with more than 40 million long-lasting insecticidal nets (LLINs) distributed between 2008 and 2013 through mass distribution campaigns, antenatal consultations, and at health clinics after completion of child vaccinations [4]. By 2014, ownership of LLINs increased to 70% of households owning one or more LLINs (with equity between urban and rural areas), while 56% of children under 5 years slept under an ITN the night prior to the survey [5]. Despite progress being made, surveys in 2013/14 showed that malaria parasite prevalence in children aged 6–59 months was still high, with a nationwide mean of 23% positive by microscopy and 34% by PCR, with the highest prevalence in Orientale province (38% by microscopy) and lowest in Nord Kivu (5% by microscopy) [5]. Under the National Malaria Control Programme (NMCP) Strategic Plan 2016–2020, the DRC seeks to ensure that at least 80% of persons at risk of malaria sleep under an LLIN [6]. LLIN replacement campaigns are ongoing, with the aim being to ensure that mosquito nets are replaced every 3 years [6, 7]. Only the pyrethroid class of insecticides are currently recommended by the World Health Organization (WHO) for LLINs [8] and pyrethroid resistance is widespread across West, East, and Southern Africa [9]. Data in central Africa, particularly DRC, is scarce despite insecticide-treated nets being the primary vector control intervention. In DRC, resistance testing from 2009 identified DDT resistance in 4 provinces and pyrethroid resistance in 3 provinces [10]. In light of the significant progress made in LLIN distribution and usage in DRC, it is important to closely monitor changes in insecticide resistance status in primary malaria vectors. Therefore, susceptibility testing was conducted in 2013 (4 provinces), 2014, 2015 and 2016 (7 provinces). Malaria vector human biting rates, biting times, and species composition were also monitored periodically, with monthly longitudinal monitoring conducted in Lodja and Kapolowe in 2016. Monitoring of malaria vector biting times is particularly important when LLINs are the main vector control tool. Nets are most effective in areas where malaria vectors bite primarily indoors late at night when the majority of people are indoors and sleeping under nets. However, changes in vector biting times have been recorded following mass distribution of LLINs [11]. As tens of millions of LLINs have been distributed in DRC in recent years it is important to determine whether vector species exhibit biting patterns that are conducive to control through LLINs.

## Methods

### Study sites

**Fig. 1:**
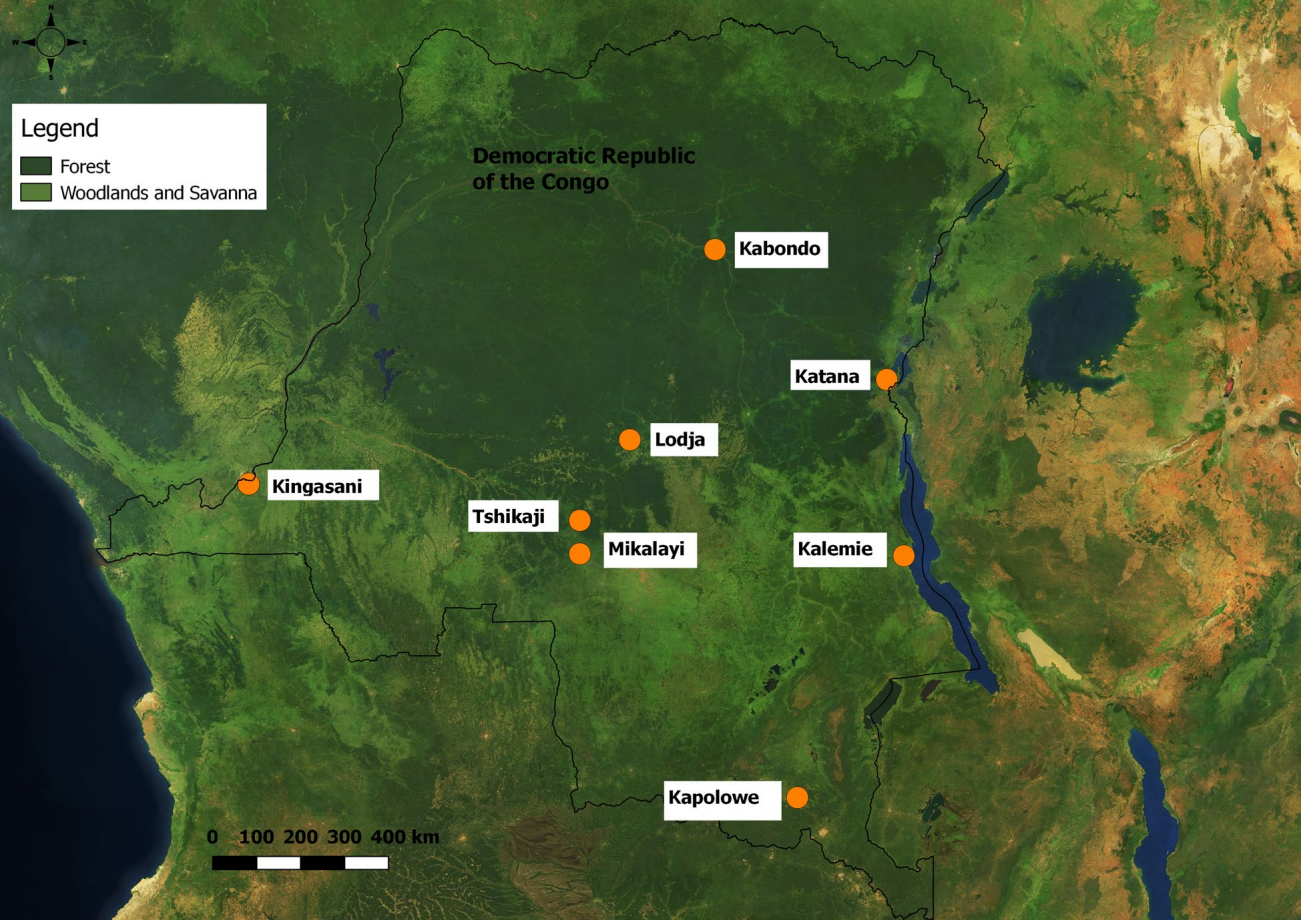
Map of Democratic Republic of the Congo indicating ecological classification and the location of eight sentinel sites for entomological monitoring

In 2013, susceptibility testing was conducted in four sentinel sites in DRC; namely Lodja, Kabondo, Tshikaji, and Kapolowe (Table 1). In 2014 Mikalayi was added to provide overlap with epidemiological sentinel site monitoring by the NMCP, and Kingasani municipality of Kinshasa was added to provide information on malaria vectors in the heavily populated capital city (> 11 million people in 2014). In 2015, to improve nationwide distribution of sites, monitoring in Tshikaji was discontinued while Kalemie and Katana in Eastern DRC were added at the request of the NMCP (Table 1). In addition to susceptibility testing, monitoring of human biting *Anopheles* was conducted in 2015 and 2016. Although several of these sites are urban and peri-urban areas, the characteristics of collection areas are often quite similar to village settings, in that traditional housing materials are commonly used and suitable larval habitats can be found. The locations of entomological monitoring sentinel sites are shown in Fig. 1.

**Table 1:** Entomological sentinel site location and frequency of susceptibility testing and trapping to determine human biting rates

Sentinel Site	Province	GPS co-ordinates	Susceptibility tests (years conducted)	Data frequency, HLC
Lodja	Sankuru	3°30′46.46″S	2013, 14, 15, 16	Monthly 2016
		23°36′2.80″E		
Kabondo	Tshopo	0°30′58.86″N	2013, 14, 15, 16	3 times per year 2015, 16
		25°13′16.23″E		
Tshikaji	Lulua	5°58′29.24″S	2013, 14	Not done
		22°27′42.65″E		
Kapolowe	Haut Katanga	10°56′23.29″S	2013, 14, 15, 16	Monthly 2016
		26°57′10.69″E		
Kingasani	Kinshasa	4°24′57.17″S	2014, 15, 16	3 times per year 2015, 16
		15°24′43.88″E		
Mikalayi	Lulua	6°1′27.06″S	2014, 15, 16	3 times per year 2015, 16
		22°19′5.70″E		
Katana	Sud Kivu	2°13′30″S	2015, 16	3 times per year 2015, 16
		28°49′53″E		
Kalemie	Tanganyika	5°55′8.59″S	2015, 16	3 times per year 2015, 16
		29°11′11.66″E		

*HLC* human landing catch

### Insecticide susceptibility tests

Larval collections were made in each study site annually between 2013 and 2016 using larval dippers. Larval sampling was done in transient, sun-exposed puddles to maximize the likelihood of sampling *Anopheles gambiae* sensu lato (s.l.). Sampling was done from several larval sites to maximize genetic variability of *An. gambiae* s.l. collected for resistance testing. The number of larval sites sampled varied from three to ten per sentinel site per year, according to availability at the time of sampling. Larvae were subsequently transported to a field insectary for rearing. Emerging adult mosquitoes were provided with sugar solution until they were used for insecticide susceptibility tests when aged 2–5 days. Insecticide susceptibility tests were conducted according to established WHO protocols using diagnostic concentrations of deltamethrin (0.05%), permethrin (0.75%), DDT (4%) and pirimiphos-methyl (0.1%) [12]. It should be noted that in 2013, WHO adopted 0.25% as the tentative diagnostic concentration for pirimiphos-methyl (while awaiting further evidence) [13].

Every year, a total of 100 *An. gambiae* s.l. per insecticide were tested at every site. Mosquitoes were exposed for 60 min in 4 replicates of 25 mosquitoes, with an additional 25 used for the negative control. After exposure, mosquitoes were transferred to clean holding tubes and provided with sugar solution. Mortality was recorded 24 h after exposure.

### Synergist bioassays

Synergist bioassays were conducted in 2015 to assess the involvement of metabolic resistance mechanisms in phenotypic resistance to permethrin. Bioassays were completed using WHO tubes lined with filter papers. The first step involved exposure of *An. gambiae* for 60 min to papers treated with 5% piperonyl butoxide (PBO). Mosquitoes were removed and exposed to papers treated with permethrin (0.75%) for an exposure time of 60 min. A total of 100 *An. gambiae* were tested in each location. In parallel, tests were conducted with permethrin (0.75%) without synergist pre-exposure. A total of 50 *An. gambiae* were exposed in tubes lined with silicone oil-treated papers as a negative control. The difference in mortality following permethrin exposure with and without pre-exposure to PBO was interpreted as the resistance due to metabolic resistance mechanisms.

The WHO has subsequently recommended using 4% PBO papers [12]. A limitation of this study is that no bioassays were done involving exposure only to synergist, to confirm that PBO did not induce lethal effects without exposure to pyrethroid. In 2016, a small sub-sample of *An. gambiae* were exposed to 5% PBO papers and mortality was < 10%.

### Human landing catches

Human landing catches (HLCs) were done primarily to determine malaria vector species composition, the location of biting (indoors or outdoors) and times of biting. Entomologists from the National Institute for Biomedical Research (INRB) in Kinshasa travelled to each sentinel site to provide technical support during three sampling periods per year: (January–March, April–June, July–September 2015 and January–February, March–April, and May–June 2016). However, for Lodja and Kapolowe sites, sampling was conducted monthly in 2016 by field based technicians that received additional training. Field technicians conducted 8 HLCs (four nights, two houses each night) during each time period. Different houses were used for each night of trapping. Four collectors were deployed per house, two for the indoor position, and two for the outdoor position. The indoor position was located in the living area of the house while the outdoor position was within five meters of the front door. The collection period was from 18:00 to 06:00, broken into two shifts of 6 h each, so that one person worked each position for half the collection period (18:00 to midnight) before being replaced by the other (midnight to 06:00). Each collector sat on a stool and exposed his lower legs and feet for mosquitoes to land on. The collector monitored mosquitoes as they landed on his legs and captured them with small glass tubes that were sealed with cotton wool. Glass tubes were then placed in a sealed bag and labelled according to the hour of collection.

The data was used to calculate the nightly human biting rate (HBR) based on eight person-nights of collection indoors and outdoors for each sampling period. Hourly data was used to plot graphs showing biting time trends. Identification of mosquitoes was done using the keys of Gillies and DeMeillon [14, 15]. The mosquito collectors conducting HLCs were recruited from the community and provided with requisite training. Collectors showing any signs of illness up to 3 weeks following collections were screened for malaria at a local health centre. There were no positive cases.

### Laboratory analysis

A sub-sample of *An. gambiae* s.l. tested in WHO susceptibility tests in 2014 were tested at INRB for the L1014F voltage-gated sodium channel (Vgsc) mutation using the protocol of Martinez-Torres et al. [16]. Kalemie and Katana were not included as sentinel sites in 2014; therefore no data is presented for these sites. Between 18 and 132 *An. gambiae* s.l. were tested for kdr genotype frequency per site. No testing for Vgsc-1014F was conducted with 2015 and 2016 samples.

A sub-sample of individually stored *An. gambiae* and *An. funestus* collected by HLC in 2015 and 2016 were analysed at INRB, Kinshasa for the presence of *Plasmodium falciparum* circumsporozoite protein (CSP) by ELISA using protocols from the MR4 Methods in *Anopheles* Research adapted from Wirtz et al. [16].

A different sub-sample of *An. gambiae* collected by HLC in 2015 were tested at the University of Notre Dame, Indiana, USA for species identification. The SINE200 protocol of Santolamazza [17], as described in the MR4 Methods in *Anopheles* Research [16], was used for distinguishing between molecular forms of *Anopheles gambiae* sensu stricto (s.s.) and *Anopheles coluzzii*. Samples from Kapolowe did not amplify while field collections from Tshikaji were discontinued in 2015.

### Climate data

Mean monthly rainfall and temperature between 1991 and 2015 for Kapolowe (Haut Katanga) and Lodja (Sankuru) was plotted into climate graphs. Mean climate data was used from an external source due to a lack of up to date, reliable meteorological data from local stations. The dataset was produced by the Climatic Research Unit (CRU) of University of East Anglia (UEA), UK and taken from The Climate Change Knowledge Portal (The World Bank Group) [18].

### Data analysis

Insecticide susceptibility results were interpreted according to WHO criteria; 98–100% mortality indicates susceptibility; 90–97% indicates possible resistance, with resistance genes to be confirmed; and mortality < 90% indicating resistance [12]. Susceptibility test mortality was corrected using Abbott’s formula if mortality in the negative control was greater than 5% and less than 20%. If mortality was greater than 20%, the test was repeated.

The difference in mortality for PBO + permethrin and permethrin only exposed mosquitoes was calculated using the Z-test for differences in proportion. Mosquito biting times are presented as polynomial regression curves. R^2^ values are presented to demonstrate the goodness of fit of the data points to the regression curve.

## Results

### Insecticide susceptibility testing

Results of WHO susceptibility tests are reported in Table 2. Susceptibility tests in 2016 indicated resistance to permethrin in five of seven provinces. Between 2013 and 2016, mortality was consistently < 50% in Kabondo, Mikalayi and Tshikaji. Between 2013 and 2016 mortality levels were relatively stable in most locations, except in Kingasani (Kinshasa) where mortality decreased from 91% (95% CI 85–97%) in 2015 to 21% (95% CI 13–29%) in 2016. In 2016 *An. gambiae* were susceptible to permethrin in Katana and Kapolowe, although resistance was documented in Kapolowe in 2013 and 2015.

**Table 2:** Percentage mortality (24 h) of *Anopheles gambiae* exposed in WHO susceptibility tests to diagnostic doses of deltamethrin (0.05%), permethrin (0.75%), DDT (4%), and pirimiphos-methyl (0.1%) at sentinel sites between 2013 and 2016

Sentinel site	Province	Deltamethrin 0.05%				Permethrin 0.75%			DDT 4%		Pirimiphos-methyl 0.1%
		2013	2014	2015	2016	2013	2015	2016	2014	2015	2015
Kabondo	Tshopo	100	99 (97–99)	85 (78–92)	76 (68–84)	27 (18–36)	25 (17–34)	12 (6–18)	17 (10–24)	37 (28–47)	100 –
Lodja	Sankuru	96 (92–99)	98 (95–99)	100	100	49 (39–59)	68 (59–77)	69 (60–78)	13 (6–20)	8 (3–13)	100 –
Kingasani	Kinshasa	n/a	99 (97–99)	97* (94–99)	100	n/a	91* (85–97)	21 (13–29)	17 (10–24)	8* (2–13)	100 –
Mikalayi	Lulua	n/a	99 (97–99)	100	88 (82–94)	n/a	30 (21–39)	36 (27–45)	42 (32–52)	15 (8–22)	100 –
Kalemie	Tanganyika	n/a	n/a	100	100	n/a	55 (45–65)	40 (30–50)	n/a	33 (24–42)	100 –
Kapolowe	Haut Katanga	95 (91–99)	99 (97–99)	100	100	31 (22–40)	53 (43–63)	100	45* (34–56)	37* (27–48 )	100 –
Katana	Sud Kivu	n/a	n/a	98 (95–99)	100	n/a	92 (87–97)	100	n/a	n/a	100 –
Tshikaji	Lulua	92 (87–97)	98 (95–100)	n/a	n/a	45 (35–55)	n/a	n/a	13 (8–24)	n/a	n/a

95% confidence intervals shown in parentheses; n = 100 for all tests except where * n = 80

In 2016, five of seven sites reported full susceptibility to deltamethrin. The only sites with confirmed resistance were Mikalayi, with resistance first reported in 2016 and Kabondo in 2015 and 2016. In Lodja, Kingasani and Kalemie, there was full susceptibility to deltamethrin despite the presence of high frequency permethrin resistance. DDT resistance was recorded in all six provinces where tests were conducted in 2014 and 2015, with mortality < 50% in every location. In all sites, *An. gambiae* were fully susceptible to pirimiphos-methyl (0.1%) when tested in 2015.

### Resistance mechanisms

**Fig. 2:**
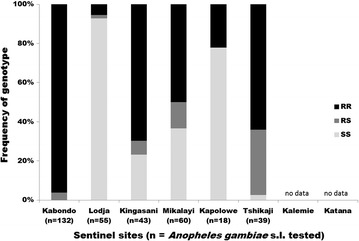
Frequency of the *Vgsc*-*1014F* mutation in *Anopheles gambiae* s.l. was at high frequency in Kabondo, Tshikaji and Kingasani but mostly wild type in Lodja. A sub-sample of *An. gambiae* s.l. were analysed following WHO susceptibility tests in 2014

Synergist tube bioassays with permethrin demonstrated a significant increase in mortality in five of seven sites following pre-exposure to PBO (Table 3). There was no significant increase in mortality following use of the synergist in Katana and Kalemie, where the frequency of resistance to permethrin was very low (mortality of 99% and 94% respectively). The mean absolute increase in mortality across all locations tested was 24%, indicating the involvement of oxidase mechanisms. The frequency of the *Vgsc*-*1014F* mutation was high (> 70%) in Kabondo, Tshikaji and Kinshasa and was low (< 20%) in Lodja and Kapolowe (Fig. 2).

**Table 3:** % Mortality of *Anopheles gambiae* in WHO tube tests with permethrin (0.75%), either with or without a 60 min pre-exposure to piperonyl butoxide (5%) treated filter papers

Sentinel site	Province	Permethrin 0.75%	PBO 5%, Permethrin 0.75%	Mean difference in mortality	*P* value
Kabondo	Tshopo	62 (53–72)	86 (79–93)	24	< 0.001
Lodja	Sankuru	79 (71–87)	100	21	< 0.001
Kingasani	Kinshasa	92 (87–97)	99 (97–99)	7	0.017
Mikalayi	Lulua	30 (21–39)	100	70	< 0.001
Kalemie	Tanganyika	94 (89–99)	96 (92–99)	2	0.516
Kapolowe	Haut Katanga	53 (43–63)	100	47	< 0.001
Katana	Sud Kivu	99 (97–99)	100	1	0.316
Mean		73 (70–76)	97 (96–98)	24	< 0.001

### Species identification

**Fig. 3:**
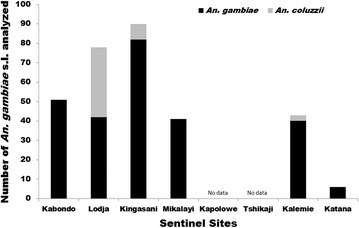
Molecular species identification using SINE 200 PCR showed mixed *An. gambiae* and *An. coluzzii* populations in Lodja but predominantly *An. gambiae* elsewhere. A sub-sample of mosquito samples were analysed collected in 2015 by HLC

Molecular species identification conducted on samples from 2015 detected only *An. gambiae* in Kabondo and Mikalayi, while in Kingasani a small proportion (8/90) of *An. coluzzii* were detected (Fig. 3). In Lodja there was a similar proportion of *An. coluzzii* and *An. gambiae*. In the Eastern site of Kalemie the majority were *An. gambiae*, but some *An. coluzzii* were detected.

### *Anopheles* human biting rates

*Anopheles gambiae* s.l. was the primary malaria vector captured in six out of seven sites. Due to study limitations including limited sample size (number of houses) and sampling frequency, human biting rates should not be considered as representative of biting risk across each province. However, there were indications of high *An. gambiae* biting risk in some sentinel sites, with particularly high biting rates recorded in Kabondo in July–September 2015 with a mean of 96 bites per person per night indoors and 68 outdoors. The human biting rate of *An. funestus* was consistently high in Mikalayi in 2015 and 2016 with a mean of > 10 bites/person/night indoors and outdoors. More details of biting rates can be found in the supplement (Additional files 1 and 2). Other potential malaria vectors, such as *Anopheles moucheti*, *Anopheles nili* and *Anopheles coustani* were found in small numbers.

**Fig. 4:**
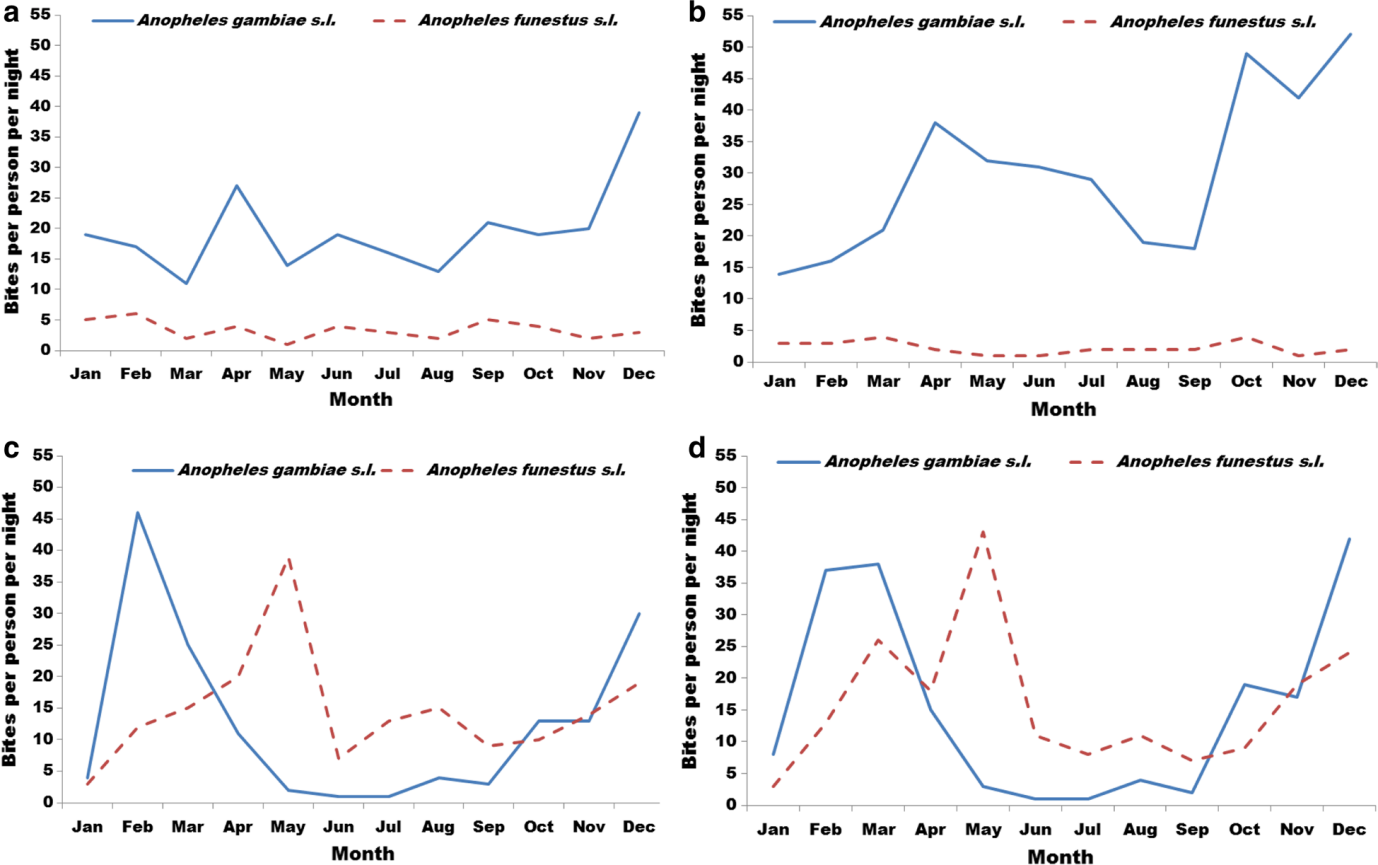
Year-round high *An. gambiae* biting rates in Lodja (Sankuru) compared with seasonal peaks of *An. gambiae* biting during the rainy season and *An. funestus* during the dry season in Kapolowe (Haut Katanga). Monthly biting rate in 2016 from HLC collections (8 houses/month) from the following locations: **a** indoors, Lodja (Sankuru). **b** Outdoors, Lodja (Sankuru). **c** Indoors, Kapolowe (Haut Katanga). **d** Outdoors, Kapolowe (Haut Katanga)

**Fig. 5:**
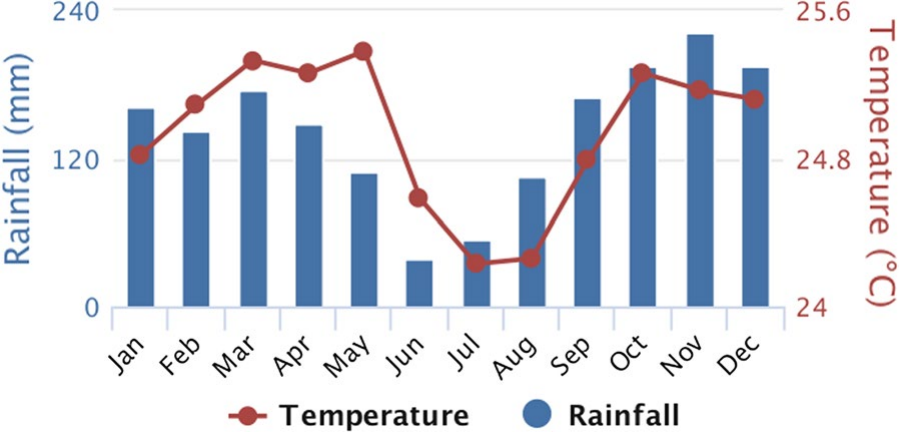
Monthly rainfall > 100 mm for 10 months (Aug–May) and warm monthly mean temperature (24–25.5 °C) in Lodja (Sankuru) provided year-round favourable conditions for *An. gambiae* s.l. Mean monthly rainfall and temperature between 1991 and 2015 for Lodja (Sankuru). The dataset was produced by the Climatic Research Unit (CRU) of University of East Anglia (UEA), UK

Monthly indoor and outdoor biting rates are presented in Fig. 4a, b for Lodja and Fig. 4c, d for Kapolowe. In Lodja, mean rainfall (1991–2015) was < 100 mm for only 2 months (June and July) of the year, with between 100 and 220 mm/month for the remaining 10 months (Fig. 5). Mean temperature was generally between 24 and 26 °C year round. Human biting rates were consistently high for *An. gambiae* and remained above 10 bites/person/night every month both indoors and outdoors. There was a biting peak in December at 40 and 52 bites/person/night indoors and outdoors (Fig. 4a, b). *Anopheles funestus* biting rates indoors and outdoors were far lower at < 5 bites/person/night year round.

**Fig. 6:**
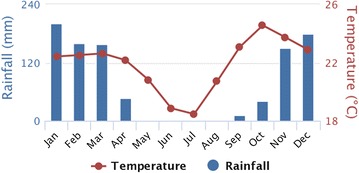
The rainy season lasted 5 months with > 100 mm rainfall (Nov–Mar) in Kapolowe (Haut Katanga) with more seasonal variation in temperature (18–25 °C) than Lodja. Mean monthly rainfall and temperature between 1991 and 2015 for Kapolowe (Haut Katanga). The dataset was produced by the Climatic Research Unit (CRU) of University of East Anglia (UEA), UK

In Kapolowe (southeastern DRC), mean rainfall was more seasonal than in Lodja (central DRC), with a pronounced dry period for 5 months between May and September, followed by a rainy season between October and April (Fig. 6). Very few (< 5/person/night) *An. gambiae* were captured in Kapolowe during the dry season between May and September, with a gradual increase in biting rates recorded between October and December. Peak human biting rates for *An. gambiae* were recorded in February at 46 and 37 bites/person/night indoors and outdoors (Fig. 4c, d). In contrast, the biting peak of *An. funestus* was later in May at 39 and 43 bites/person/night followed by considerable biting throughout the dry season at between 5 and 15 bites/person/night between June and September. Biting trends for both *An. gambiae* and *An. funestus* were very similar indoors and outdoors.

### Biting times of *Anopheles gambiae* and *Anopheles funestus*

**Fig. 7:**
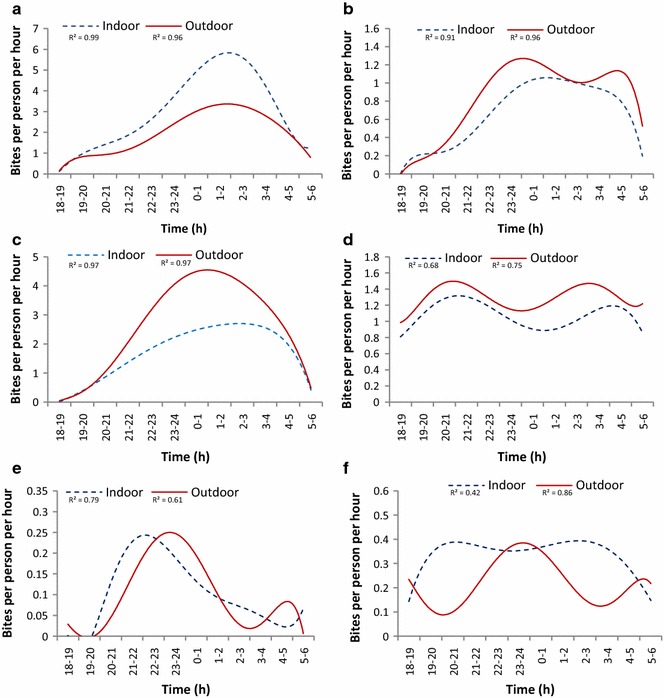
Biting times of *An. gambiae* s.l. in six sentinel sites across DRC. **a** Kabondo, Tshopo province (2015–16), n = 1671 indoors, 1075 outdoors. **b** Kingasani, Kinshasa (2015–16), n = 356 indoors, 455 outdoors. **c** Lodja, Sankuru province (2016), n = 1870 indoors, 2878 outdoors. **d** Kapolowe, Haut Katanga province (2016), n = 1219 indoors, 1483 outdoors. **e** Katana, Sud Kivu province (2015–16), n = 58 indoors, 54 outdoors. **f** Kalemie, Tanganyika province (2015–16), n = 187 indoors, 127 outdoors. *R^2^ values are presented to demonstrate the goodness of fit of the data points to the regression curve

Biting times of *An. gambiae* were similar in Kabondo, Kingasani and Lodja, with the majority of biting occurring late at night between 22:00 and 04:00 with similar trends both indoors and outdoors (Fig. 7a–c). In Kapolowe, considerable biting occurred early in the evening both indoors and outdoors from 19:00 and remained fairly consistent throughout the night, except for two minor peaks between 20:00–22:00 and 02:00–04:00 (Fig. 7d). In Katana, there is an indication of a biting peak occurring relatively early in the evening between 21:00 and 01:00; however the sample size was small (Fig. 7e). In Kalemie, the sample size was also relatively small and there were no clear trends (Fig. 7f).

**Fig. 8:**
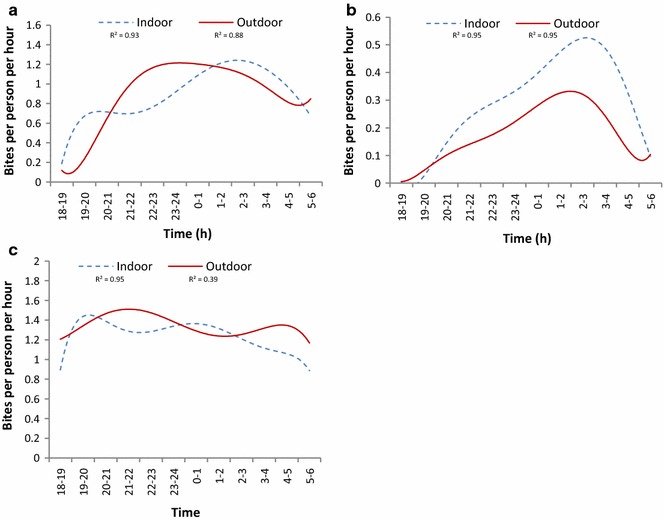
Biting times of *An. funestus* s.l. in three sentinel sites in DRC. **a** Mikalayi, Lulua province (2015–16), n = 493 indoors, 503 outdoors. **b** Lodja, Sankuru province (2016), n = 320 indoors, 200 outdoors. **c** Kapolowe, Haut Katanga province (2016), n = 1400 indoors, 1536 outdoors. *R^2^ values are presented to demonstrate the goodness of fit of the data points to the regression curve

In Mikalayi, *An. funestus* biting rates were relatively high by 21:00 in the evening and continued to increase until a peak was recorded between 22:00 and 04:00 (Fig. 8a). In Lodja, *An. funestus* biting rates were low in the early evening and gradually increased to a peak between 23:00 and 04:00 (Fig. 8b). In Kapolowe, considerable *An. funestus* biting took place between 18:00 and 19:00 and continued throughout the night until 6 a.m. (Fig. 8c).

### *Plasmodium falciparum* sporozoite rates for *Anopheles gambiae* and *Anopheles funestus*

Sporozoite rates for all seven sites are presented in Table 4. The mean sporozoite rate across all seven sites was 4.3% (95% CI 3.4–5.2) for *An. gambiae* and 3.3% for *An. funestus* (95% CI 1.3–5.3). While sampling design limitations do not allow for direct comparison between sentinel sites, sporozoite rates were relatively high at all sites. The lowest sporozoite rate for *An. gambiae* was in Katana at 2.8% (95% CI 1.1–4.6) and for *An. funestus* in Mikalayi at 2.0% (95% CI 0.1–4.3) and Lodja at 0% (although only 24 samples were tested). Sporozoite rates were also high in municipalities of major cities such as Kingasani in Kinshasa at 4.6% (95% CI 3.4–5.2) for *An. gambiae*.

**Table 4:** *Plasmodium falciparum* sporozoite positive rates for *An. gambiae* and *An. funestus* collected by human landing catch in seven sentinel sites of DRC

Sentinel site	Province	Species	Number tested	Number sporozoite positive	% Sporozoite positive (95% CI)
Kabondo	Tshopo	*An. gambiae*	219	8	*3.7* (1.2–6.1)
Lodja	Sankuru	*An. gambiae*	307	9	*2.9* (1.0–4.8)
		*An. funestus*	24	0	*0*
Kingasani	Kinshasa	*An. gambiae*	324	15	*4.6* (2.3–6.9)
Mikalayi	Lulua	*An. gambiae*	330	17	*5.2* (2.8–7.5)
		*An. funestus*	149	3	*2.0* (0.1–4.3)
Kalemie	Tanganyika	*An. gambiae*	203	13	*6.4* (3.0–9.8)
		*An. funestus*	9	1	*11.1* (0.1–31.6)
Kapolowe	Haut Katanga	*An. gambiae*	127	8	*6.3* (2.1–10.5)
		*An. funestus*	50	4	*8.0* (0.5–15.5)
Katana	Sud Kivu	*An. gambiae*	351	10	*2.8* (1.1–4.6)
		*An. funestus*	70	2	*2.9* (0.1–6.8)
Total		*An. gambiae*	1861	80	*4.3* (3.4–5.2)
		*An. funestus*	302	10	*3.3* (1.3–5.3)

## Discussion

Effective management of insecticide resistance requires an understanding of the dynamics and mechanisms driving resistance. Despite the vast size of the country, some general trends appear; namely widespread *An. gambiae* resistance to DDT and permethrin, but susceptibility to deltamethrin in most sites. The finding of high frequency DDT resistance in all 7 sites supports findings from 2009 showing DDT resistance in Kinshasa, Bolenge, Kimpese and Katana [10]. The Stockholm Convention on Persistent Organic Pollutants stipulates that countries can use DDT for vector control when safe, effective and affordable alternatives are not locally available [19]. However, there is little prospect of DDT use for malaria control in DRC because of widespread vector resistance.

In 2009, Kanza et al. [10] reported that *An. gambiae* were susceptible to permethrin in Kinshasa; however, Bobanga et al. [20] reported some resistance in 2010. In 2016 permethrin resistance was recorded at relatively high frequency in Kabondo, Lodja, Mikalayi, Kalemie and Tshikaji. Conversely, *An. gambiae* in Lodja, Kinshasa and Kalemie were susceptible to deltamethrin despite permethrin resistance being confirmed. The frequency of resistance is known to vary within the pyrethroid class depending on the specific resistance mechanisms involved [21]. In south-eastern Côte d’Ivoire, *An. gambiae* were found to be resistant to permethrin (40% mortality) and alphacypermethrin (64% mortality), but largely susceptible to deltamethrin (98% mortality) [22]. A similar result was observed in N’Djamena, Chad where *Anopheles arabiensis* were resistant to permethrin (54% mortality), but had a low frequency of resistance to deltamethrin (91% mortality) [23]. The metabolic detoxification enzyme CYP6P4 was over-expressed in *An. arabiensis* from Chad and was believed to be the primary resistance mechanism. Expression studies demonstrated that CYP6P4 readily metabolizes permethrin but is unable to metabolize deltamethrin, apparently explaining the finding of near susceptibility to deltamethrin [24]. In DRC, the increase in mortality when *An. gambiae* were pre-exposed to PBO implicates mixed function oxidase (MFO) resistance mechanisms. In several sites, including Lodja, Mikalayi and Kapolowe, full susceptibility was restored after exposure to PBO, which may indicate that elevated MFOs are the primary resistance mechanism. However, in other locations such as Kabondo, bioassays with a synergist did not restore susceptibility and the frequency of the *Vgsc*-*1014F* allele was high, indicating that multiple resistance mechanisms are involved. The *Vgsc*-*1014F* allele is associated with resistance to both type-1 and type-2 pyrethroids and does not appear to explain why there was a greater frequency of resistance to permethrin than deltamethrin in several sites [25, 26].

It is important to monitor specific primary resistance mechanisms in DRC so that heterogeneity of resistance can be taken into account for resistance management. To guide future LLIN distribution campaigns in DRC, it is important to determine whether deltamethrin LLINs provide greater control than permethrin LLINs in areas of permethrin resistance, but where deltamethrin susceptibility remains. Despite high frequency permethrin resistance being recorded in several sites of DRC, the high protective effect of permethrin, linked to its spatial repellent effect, is likely to continue to provide some degree of protection [27]. A recent multi-country study coordinated by the WHO in Benin, Cameroon, India, Kenya and Sudan provided evidence that LLINs continued to provide personal protection against malaria in areas with pyrethroid resistance [28]. However, the WHO study did not measure the impact of pyrethroid resistance on the mass mosquito killing effect, which is an important component of community protection.

Full susceptibility to pirimiphos-methyl in seven sentinel sites was documented in 2015 using a concentration of 0.1%, which is lower than the 0.25% tentatively recommended by the WHO [13]. Actellic^®^ 300CS (Syngenta, Switzerland) was utilized for IRS in 36,000 houses within the Tenke-Fungurume Mining Concession (Lualaba province) in 2015 and 2016 [6]. The finding of full susceptibility in all 7 sentinel sites indicates that expansion of IRS with Actellic CS could be a potential vector control strategy, where logistically feasible.

In human landing catches *An. gambiae* and *An. funestus* were the most commonly captured malaria vectors. Molecular species identification demonstrated the presence of sympatric populations of *An. gambiae* s.s. and *An. coluzzii* in Lodja and Kingasani (Kinshasa). Earlier studies also documented the presence of sympatric populations of *An. gambiae* s.s. and *An. coluzzii* in Kinshasa [20, 29]. However, Bobanga et al. reported only *An. gambiae* s.s. in Lodja (2004) and Kapolowe (2010). The finding of three *An. coluzzii* in Kalemie is just within the predicted easterly distribution limits according to species distribution models [30]. It will be important to conduct molecular species identification in future studies to determine whether there are differences in resistance frequency and mechanisms.

In the majority of sentinel sites *An. gambiae* and *An. funestus* exhibited ‘classic’ biting rhythm with most biting risk occurring late at night. However, in Kapolowe, significant biting of *An. gambiae* and *An. funestus* was observed early in the evening both indoors and outdoors, before people are likely to be protected by LLINs. *Anopheles gambiae* biting early in the evening were reported in several countries in Africa, including Kenya and Uganda [11, 31, 32]. Changes in vector biting behavior have been associated with prolonged use of insecticides. In Papua New Guinea a shift in median *Anopheles farauti* biting times to early morning was recorded several years after mass LLIN distribution [33]. Changes in biting behaviour were also reported for *An. funestus* in Benin and Senegal following LLIN universal coverage campaigns [34, 35]. However, a review by Gatton et al. noted that while there are numerous examples demonstrating shifts in vector biting behaviour after IRS or LLIN campaigns, this is certainly not the case everywhere [36].

Monitoring monthly biting trends in Lodja and Kapolowe demonstrated important seasonal dynamics that are vital when considering vector control interventions. In Kapolowe, the malaria transmission season was extended by *An. funestus* due to larval sites consisting of more permanent water bodies such as lake margins, which are less rainfall dependent than *An. gambiae* [37].

Urbanization is generally associated with decreased risk of malaria infection, due to reduction in suitable larval habitats and socio-economic changes. Despite a growing urban population of more than 11 million people in Kinshasa, *An. gambiae* biting rates and sporozoite rates in the municipality of Kingasani remained high, although lower than the 7.8% sporozoite rate recorded in 1993 by Coene [38]. Ferrari et al. found that malaria prevalence in children (age 6–59 months) was 25% (95% CI 18.3–32.7) in Kingasani in 2011, compared to a mean of 11.9% across Kinshasa [39]. The presence of flood zones from rivers used for urban agriculture and a relatively long rainy season mean that *An. gambiae* breeding sites are still plentiful in parts of Kinshasa.

## Conclusion

The high biting rates and sporozoite rates encountered across most sentinel sites are a reminder that multiple control and prevention measures are likely to be needed in meso- and hyper-endemic areas of DRC. The finding of widespread resistance to permethrin in DRC is concerning and LLINs with non-pyrethroid insecticides should be evaluated.

## Additional files

**Additional file 1.** Biting rates (bites per person per night) of *An. gambiae* based on human landing catches (HLC) conducted indoors and outdoors from sentinel sites in Democratic Republic of Congo in 2015 and 2016.

**Additional file 2.** Biting rates (bites per person per night) of *An. funestus* based on human landing catches (HLC) conducted indoors and outdoors from sentinel sites in Democratic Republic of Congo in 2015 and 2016.
