# Supplementary material for: Nationwide insecticide resistance status and biting behaviour of malaria vector species in the Democratic Republic of Congo
Source: Malar J. 2018 Mar 27;17:129. doi: 10.1186/s12936-018-2285-6 (PMC5870394; doi:10.1186/s12936-018-2285-6)
Supplement: Supplementary file 1 — Additional file 1. Biting rates (bites per person per night) of An. gambiae based on human landing catches (HLC) conducted indoors and outdoors from sentinel sites in Democratic Republic of Congo in 2015 and 2016. [file 12936_2018_2285_MOESM1_ESM.docx]

**Additional files**

**Additional file 1: Biting rates (bites per person per night) of *An. gambiae* based on human landing catches (HLC) conducted indoors and outdoors from sentinel sites in Democratic Republic of Congo in 2015 and 2016.**

| **Period** | **Location** | **Kabondo, Tshopo** | **Kingasani, Kinshasa** | **Mikalayi, Lulua** | **Kalemie, Tanganyika** | **Katana, Sud Kivu** |
| --- | --- | --- | --- | --- | --- | --- |
| **2015 *An. gambiae*** | | | | | | |
| Jan to Mar | Indoor | 13 | 4 | 12 | 0.4 | 0.3 |
|  | Outdoor | 3 | 8 | 10 | 1 | 0.4 |
| Apr to Jun | Indoor | 9 | 7 | 5 | 2 | 1 |
|  | Outdoor | 5 | 3 | 8 | 2 | 1 |
| Jul to Sep | Indoor | 96 | 2 | 13 | 3 | 1 |
|  | Outdoor | 68 | 5 | 16 | 2 | 1 |
| 2015 mean | Indoor | 39 | 4 | 10 | 2 | 1 |
|  | Outdoor | 25 | 5 | 11 | 2 | 1 |
| **2016 *An. gambiae*** | | | | | | |
| Jan to Feb | Indoor | 18 | 2 | 6 | 5 | 2 |
|  | Outdoor | 14 | 7 | 1 | 3 | 2 |
| Mar to Apr | Indoor | 68 | 12 | 5 | 7 | 2 |
|  | Outdoor | 39 | 11 | 4 | 3 | 2 |
| May to Jun | Indoor | 6 | 14 | 1 | 7 | 2 |
|  | Outdoor | 6 | 19 | 1 | 5 | 1 |
| 2016 mean | Indoor | 30 | 9 | 4 | 6 | 2 |
|  | Outdoor | 20 | 12 | 2 | 4 | 1 |
